# Unaltered Angiogenesis-Regulating Activities of Platelets in Mild Type 2 Diabetes Mellitus despite a Marked Platelet Hyperreactivity

**DOI:** 10.1371/journal.pone.0162405

**Published:** 2016-09-09

**Authors:** Xinyan Miao, Wei Zhang, Zhangsen Huang, Nailin Li

**Affiliations:** Karolinska Institutet, Department of Medicine-Solna, Clinical Pharmacology, Karolinska University Hospital-Solna, 171 76, Stockholm, Sweden; University of Kentucky, UNITED STATES

## Abstract

Type 2 diabetes mellitus (T2DM) is associated with platelet dysfunction and impaired angiogenesis. Aim of the study is to investigate if platelet dysfunction might hamper platelet angiogenic activities in T2DM patients. Sixteen T2DM patients and gender/age-matched non-diabetic controls were studied. Flow cytometry and endothelial colony forming cell (ECFC) tube formation on matrigel were used to assess platelet reactivity and angiogenic activity, respectively. Thrombin receptor PAR1-activating peptide (PAR1-AP) induced higher platelet P-selectin expression, and evoked more rapid and intense platelet annexin V binding in T2DM patients, seen as a more rapid increase of annexin V^+^ platelets (24.3±6.4% vs 12.6±3.8% in control at 2 min) and a higher elevation (30.9±5.1% vs 24.3±3.0% at 8 min). However, PAR1-AP and PAR4-AP induced similar releases of angiogenic regulators from platelets, and both stimuli evoked platelet release of platelet angiogenic regulators to similar extents in T2DM and control subjects. Thus, PAR1-stimulated platelet releasate (PAR1-PR) and PAR4-PR similarly enhanced capillary-like network/tube formation of ECFCs, and the enhancements did not differ between T2DM and control subjects. Direct supplementation of platelets to ECFCs at the ratio of 1:200 enhanced ECFC tube formation even more markedly, leading to approximately 100% increases of the total branch points of ECFC tube formation, for which the enhancements were also similar between patients and controls. In conclusion, platelets from T2DM subjects are hyperreactive. Platelet activation induced by high doses of PAR1-AP, however, results in similar releases of angiogenic regulators in mild T2DM and control subjects. Platelets from T2DM and control subjects also demonstrate similar enhancements on ECFC angiogenic activities.

## Introduction

Type 2 diabetes mellitus (T2DM) is associated with a high risk of cardiovascular complications due to diabetic angiopathy [1, 2]. The development of diabetic angiopathy and atherosclerosis involves multiple mechanisms. Apart from inflammatory and thrombotic mechanisms, dysfunction of angiogenesis in diabetic patients has also been recognized as an important contributor to diabetic angiopathy [3], which is manifested by reduced tissue regeneration and organ reparation. The causes leading to aberrant angiogeneses in T2DM are mutiple, but have not been fully understood.

Platelets from T2DM patients are hyperactive [2, 4]. There are more circulating activated platelets and platelet-leukocyte aggregates in T2DM patients [4, 5]. Diabetic platelets are more reactive, seen as enhanced platelet secretion [5, 6] and aggregation upon *in vitro* stimuli [7]. Moreover, platelets store and release a number of angiogenic factors [8], and contribute importantly to angiogenesis [9]. It has also been shown that platelets store pro-angiogenic regulators and anti-angiogenic regulators in separate α-granules [8, 10], and that different stimuli, namely thrombin via PAR1 receptor/ADP via P2Y12 receptor and thrombin via PAR4 receptor/thromboxane A_2_ (TxA_2_) via TP receptor, induce selective releases of pro- and anti-angiogenic regulators of platelets [8, 10–12], respectively. We have recently shown that platelets exert their angiogenic regulatory effects through not only released/soluble mediators but also cell membrane glycoproteins [13, 14]. Giving the importance of platelets in angiogenesis and the impacts of angiopathy on T2DM cardiovascular complications, there is a need to investigate if and how T2DM alters platelet angiogenic activities. Albeit having been studied before [15], there is a paucity of information with regard to potential alternations of platelet angiogenic activities in T2DM.

Diabetic angiopathy involves dysfunctions of endothelial cells (ECs). Thus, ECs of T2DM patients are linked to reduced production of antithrombotic prostaglandin I_2_ (PGI_2_) and nitric oxide (NO) [16], as well as overproduction of pro-inflammatory reactive oxygen species (ROS) and reactive nitrogen species (RNS) [17]. Diabetic ECs have a reduced endothelial proliferative/regenerative potential, impaired barrier function, and an increased adhesiveness to other circulating cells [18]. Moreover, circulating endothelial colony forming cells (ECFCs, also refered as endothelial progenitor cells) have now been recognized as an important source for endothelial regeneration and reparation of the injured vessel wall [19]. T2DM patients are known to have a decreased number and impaired functionality (e.g., with a reduced propensity of capillary-like network formation in vitro) of circulating ECFCs [20, 21]. It has also been shown that ECFC recruitment at the sites of arterial injury is impaired in diabetic mice [21]. Hence, it is of interest to study how T2DM platelets can regulate angiogenic activities of ECFCs.

The present study was aimed to test the hypothesis that diabetic platelet dysfunction in T2DM patients might alter angiogenic properties of platelets, which would subsequently hamper their capacity to regulate angiogenic activities of ECFCs. Our results showed that platelets from T2DM patients were hyperreactive, as evidenced by enhanced platelet P-selectin expression and by more rapid and more intense increases of platelet phosphotidylserine exposure (annexin V binding). Platelets of those mild T2DM patients had, however, similar angiogenic activities (angiogenic regulator release and platelet-regulated capillary-like network formation of ECFCs) as compared to those of age/gender-matched controls.

## Materials and Methods

### Study subjects

Type 2 diabetes mellitus (T2DM) and nondiabetic control subjects (n = 16 for both) gave informed and written consent to participate in this study, which was approved by the Ethics Committee of Karolinska Institutet. Demographic data of diabetic and control subjects are shown in the Table 1.

**Table 1:** Demographic data and medical treatments of the study subjects.

	T2DM Patients	Non-diabetic controls
	(n = 16)	(n = 16)
Age	62±2	61±2
Gender: Female/Male	7/9	7/9
Body mass index	26.7±0.7*	23.9±0.7
DM duration, median(range)	9 (1–16)	––
Blood glucose (mM)	8.5±0.8*	5.0±0.2
HbA1c %†	6.3±0.2*	4.6±0.1
Hypoglycemic treatments		
Biguanides	11	––
Sulphonylureas	1	––
glitazones	1	––
insulin	3	––
Aspirin	4	2
Statins	9	1
Beta blocker	2	1
Calcium antagonist	3	––
ACE inhibitor	3	––

* P<0.05, as compared to non-diabetic controls.

^†^ HbAc1 was measured using the Mono-S method with a reference value of <5.2%.

### Reagents

Acid-soluble thrombin receptor PAR1 activating peptide (PAR1-AP) was from Calbiochem (Darmstadt, Germany). Water-soluble PAR1-AP and PAR4 activating peptide (PAR4-AP), prostaglandin I_2_ (PGI_2_), and cultured cell detach solution (0.01% trypsin/5 mM EDTA) were purchased from Sigma (St Louis, MO, USA). Platelets were identified by the fluorescein isothiocyanate (FITC)-conjugated CD42a MAb Beb 1 (Becton Dickinson; San Jose, CA, USA). Platelet P-selectin expression was determined by R-phycoerythrin (PE)-CD62P MAb, and platelet phosphotidylserine (PS) exposure was monitored by PE-conjugated annexin V (both from Becton Dickinson). Immunoassay kits monitoring platelet release of vascular endothelial growth factor (VEGF), platelet-derived growth factor (PDGF, BB type), platelet factor 4 (PF4), and thrombospondin-1 (TSP-1) were from R&D Systems Ltd (Abingdon, UK). Endothelial culture media (EBM-2 Basal Medium and the EGM-2 SingleQuots kit) and fetal bovine serum (FBS) were purchased from Lonza (Basel, Switzerland). Matrigel ^™^ Matrix was from Becton Dickinson.

### Blood sample handling and washed platelet preparation

Venous blood was collected by venepuncture without stasis, using siliconized vacutainers containing 1/10 volume of 129 mM trisodium citrate or 200 μg/ml recombinant hirudin (Ciba Geigy, Base, Switzerland). Platelet rich plasma (PRP) was prepared by centrifugation (150 ×*g*, 15 min, 22°C), and the PRP was further centrifuged at 330 ×g for 15 min after supplemented with 0.1 volume of Acid-Citrate-Dextrose (ACD) buffer and PGI_2_ (0.1 μg/ml, final concentration). The platelet pellet was suspended in Tyrode’s Hepes buffer and adjusted the concentration to 1×10^8^/ml or 1×10^9^/ml.

### Dynamics of platelet activation

Hirudized whole blood was added into the pre-warmed curvettes. The blood samples were then incubated in the presence of vehicle or 10 μM PAR1-AP (final concentration) during 8 min at 37°C. At the time points of 0, 2, 4, 6, and 8 min, an aliquot of 2 μl blood was added to the binding buffer (NaCl 140 mM, CaCl2 2.5 mM, HEPES 10 mM, pH7.4) containing PE-Annexin V and FITC-CD42a MAb. After 20 min incubation in dark at room temperature, the staining was terminated by further dilution with 800 μl binding buffer. Thereafter, annexin V-binding of single platelets was analysed by flow cytometry.

### Flow cytometry

Flow cytometric platelet analyses were performed as previously described [22]. Briefly, 5 μl whole blood or washed platelets were added to 45 μl HEPES-buffered saline (pH7.4) containing appropriately diluted antibodies and without or with a platelet agonist. The samples were incubated at room temperature in dark for 20 min. Afterwards, the samples were midly fixed with 0.5% (v/v) formaldehyde saline, and analysed with a Beckman-Coulter FC500 flow cytometer (Beckman-Coulter Corp., Hialeah, FL). Platelets were gated according to their charateristics of light scattering signals, and the gating was confirmed by FITC-CD42a staining (>98% positive). Platelet P-selectin expression or platelet annexin V binding were monitored and reported as the percentages and mean fluorescence intensity (MFI) of positive cells in the total platelet population.

### Preparation of platelet releasates

After the recovery of platelets reactivity (30 min, 22°C), platelets were stimulated with vehicle (Tyrode’s Hepes buffer), PAR1-AP (80 μM), or PAR4-AP (100 μM) for 10 min at 37°C. PAR1-AP and PAR4-AP concentrations were settled after a careful evaluation, in which both agonists provoked intense platelet activation to the same extent, and induced optimal platelet release. Platelet activation was terminated by an ice bath, and the samples were then centrifuged at 1000 ×g for 10 min at 4°C. The supernatants were collected and centrifuged again at 15000 ×g for 10 min at 4°C. Afterwards, the releasates were aliquoted and stored at -80°C.

### Immunoassays

Quantikine^®^ ELISA kits detecting platelet-derived growth factor (PDGF), vascular endothelial growth factor (VEGF), platelet factor 4 (PF4), and thrombosponding-1 (TSP1) were purchased from R&D Systems. The immunoassays of platelet releasates were carried out according to the manufacturer's instructions. The standards and the samples were run in duplicates.

### In vitro tube formation assay on a Matrigel plate

Human endothelial colony forming cells (ECFCs) were cultivated from peripheral blood mononuclear cells in our laboratory, and the culture procedure and ECFC phenotypings have been described in details [13, 14]. ECFCs were cultured in endothelial basal medium-2 (EBM-2) supplemented with 10% FBS and SingleQuots (growth factors, cytokines, and supplements) in a humidified cell culture incubator with 5% CO_2_ at 37°C. After the cultured cells had reached approximately 80% confluent, the cells were harvested and re-suspended in EBM-2 basal medium at the concentration of 1–1.25×10^5^ cells/ml.

Tube formation assay was performed on the Matrigel-coated 96-well culture plates. Matrigel was thawed at 4°C overnight, placed 50 μl per well in a 96-well plate, and incubate the plate at 37°C for 1 h to allow matrix gel solidification. Afterwards, 10^4^ ECFCs in 100 μl EBM-2 basic medium without or with platelets (ECFC:platelet ratio at 1:200) or 10% platelets releasates were added into each matrix gel-coated well and incubated at 37°C for 6 h. Formation of capillary-like tubular structures was visualized under an Olympus CKX41 inverted light microscope (with a 10× objective) equipped with a Nikon D5100 camera. Five representative fields (i.e., up-left, lower-left, central, up-right, and lower-right sites of a well) from each well were photographed. Branch points, total tube length, loop numbers, and cell-covered area of the capillary structure were assessed using the WimTube tube formation image analysis platform (Wimasis image analysis, Munich, Germany).

### Data presentation and statistics

Data are presented as mean±SEMs. Comparisons were performed with Student paired *t* test and/or repeated measurements ANOVA using GraphPad Prism 5 (GraphPad Software; CA, USA). P<0.05 was considered to indicate significance.

## Results

### Demongraphic data of the study subjects

The T2DM patients had elevated levels of blood glucose and HbA1c, and the patients also had a higher BMI as compared to the age- and gender-matched non-diabetic controls (Table 1). Twelve T2DM patients received oral hypoglycemic drug treatments, of whom three patients also received insulin treatments, while four patients were on diet only. Four T2DM patients were on a low-dose aspirin treatment, while two control subjects also took aspirin.

### Platelet hyperactivity in T2DM patients

**Fig 1:**
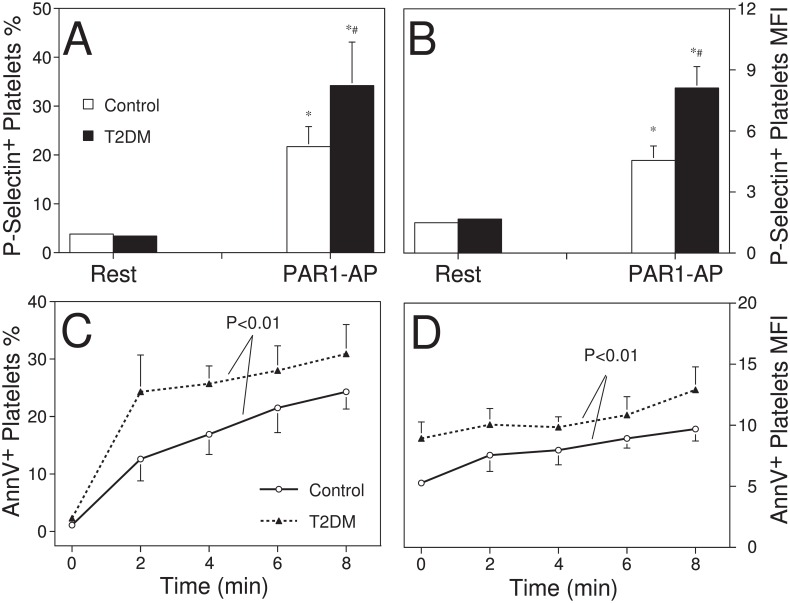
Platelets are hyperreactive in T2DM patients. Panels A and B: Hirudinized whole blood of non-diabetic (open bars) and T2DM subjects (filled bars) was incubated in the presence of PE-conjugated anti-P-selectin MAb and in the absence or presence of 4 μM PAR1-AP for 20 min at 22°C. Platelet P-selectin expression was determined by flow cytometry. The data plotted are mean±SEM the percentages (panel A) and mean fluorescence intensities (panel B) of P-selectin positive platelets in the total platelet population; n = 8. *P<0.05 vs unstimulated samples; #P<0.05 vs non-diabetic controls. Panels C and D: Hirudinized whole blood was incubated at 37°C in the presence of 10 μM PAR1-AP during 8 min. At the time points of 0, 2, 4, 6, and 8 min, blood aliquots were added into the binding buffer containing PE-conjugated annexin V and FITC-CD42a MAb, and incubated for 20 min at 22°C. Thereafter, annexin V-binding of single platelets was analysed by flow cytometry. Mean±SEMs of the percentages (panel C) and mean fluorescence intensities (panel D) of annexin V binding positive platelets are plotted, n = 7; P value of repeated measurements ANOVA indicates the difference between non-diabetic (open circles and solid line) and T2DM subjects (filled triangles and dash line).

Fig 1A shows that the basal level of platelet P-selectin expression in whole blood was 3.4±0.4% in T2DM patients, similar to that of control subjects (3.8±0.5%; *P* = 0.80). However, upon stimulation with submaximal concentration of PAR1-PA at 4 μM, platelet P-selectin expression was increased more markedly in T2DM patients (34.2±8.9%) than in non-diabetic controls (21.7±4.1%; *P*<0.05). When mean fluorescence intensities (MFIs) of P-selectin positive platelets were analysed (Fig 1B), it was found that platelet P-selectin MFIs in unstimulated samples were similar between the two groups. PAR1-PA stimulation significantly increased the intensity of platelet P-selectin expression, as reflected by elevated MFIs, and the enhancement was more marked in the patients than in non-diabetic controls.

To demonstrate the dynamics of platelet activation, PE-conjugated Annexin V was also used to track platelet surface expression of negatively-charged phospholipids, mainly phosphotidylserine (PS), in response to 10 μM PAR1-AP stimulation during 8 minutes. As can be seen in Fig 1C and 1D, unstimulated platelets of both T2DM patients and control subjects had a small population of annexin V positive platelets, indicating a low level of PS exposure. Notably, basal annexin V binding in T2DM patients was higher than that of control subjects, both in terms of annexin V binding positive percentages (2.3±0.4% vs 1.1±0.3%; P<0.05) and MFI (8.92±1.35 vs 5.27±0.37; P<0.05). The intense platelet activation induced by 10 μM PAR1-AP stimulation, which increased P-selectin expression positive platelets to >95%, provoked significant increases of platelet annexin V binding in both patients and controls. Fig 1C shows that the positive percentages of platelet annexin V binding/PS exposure in T2DM patients were much more marked and rapid. The latter was evidenced by that more than 75% increment of annexin V binding had already been reached by 2 min, as compared to that of less than 50% in the control subjects. Moreover, the stimulus also induced a greater increase of annexin V binding in T2DM patients (30.9±5.1% at 8 min) than in the controls (24.3±3.0%; P<0.01). Fig 1D demonstrates that PAR1-AP stimulation also enhanced platelet annexin V binding/PS exposure intensities over time, and that higher levels of platelet annexin V binding/PS exposure in T2DM patients were maintained throughout the observation.

### Platelet release of angiogenic regulators upon thrombin receptor activation

Activated platelets release a number of angiogenic regulators, such as VEGF, PDGF, PF4, TSP-1, and bFGF, which can regulate the ECFC tube formation. To investigate whether there is an altered release pattern of platelet angiogenic regulators in T2DM patients, we ran a set of ELISAs for quantifying platelet-released VEGF, PDGF, PF4 and TSP-1 upon PAR1-AP and PAR4-AP stimulation. Of note, to be able to use the platelet releasates (PRs) for the experiments of PR-regulated ECFC tube formation, we chose to use water-soluble PAR1-AP and PAR4-AP. Platelet activating potentials of these peptides had been carefully titrated, and it was found that water-soluble PAR1-AP at 80 μM and PAR4-AP at 100 μM induced a similar extent of platelet activation, which similarly increased platelet P-selectin expression to approximately 95% in washed platelets prepared from blood samples of non-diabetic subjects.

**Fig 2:**
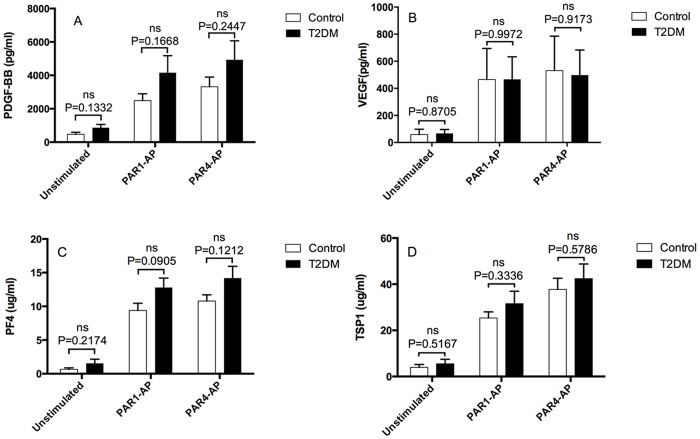
PAR1-AP and PAR4-AP induce similar platelet releases of angiogenic regulators in T2DM and non-diabetic control subjects. Washed platelets were prepared from citrated whole blood obtained from control (open bars) and T2DM subjects (filled bars), and were resuspended in Tyrode’s Hepes buffer at 10^9^/ml. The cell suspension was challenged with vehicle/Tyrode’s Hepes buffer, PAR1-AP (80 μM), or PAR4-AP (100 μM) at 37°C for 10 min. The levels of PDGF-BB (panel A), VEGF (B), PF4 (C) and TSP1 (D) in the supernatants from above samples were analysed by corresponding ELISA kits from R&D Systems. Data presented are mean±SEM from 5-matched pairs of control (grey bars) and T2DM subjects (filled bars). P values plotted were obtained by Student’s paired t test.

Fig 2 shows that PAR1-AP (80 μM) and PAR4-AP (100 μM) both markedly enhanced platelet release of angiogenic regulators in T2DM and control subjects. Both agonists tended to induced slightly greater releases of PDGF (Fig 2A) and PF4 (C) in T2DM patients than in control subjects (P values ranging from 0.09 to 0.24), whilst they induced similar releases of VEGF (B) and TSP-1 in patients and controls. Furthermore, PAR1-AP tended to induce slightly lower release of PDGF (A) and TSP-1 (D) as compared to PAR4-AP, but both activating peptides enhanced platelet release of VEGF (B) and PF4 (C) to a similar extent.

### Effects of platelet releasates on tube formation of endothelial colony forming cells

**Fig 3:**
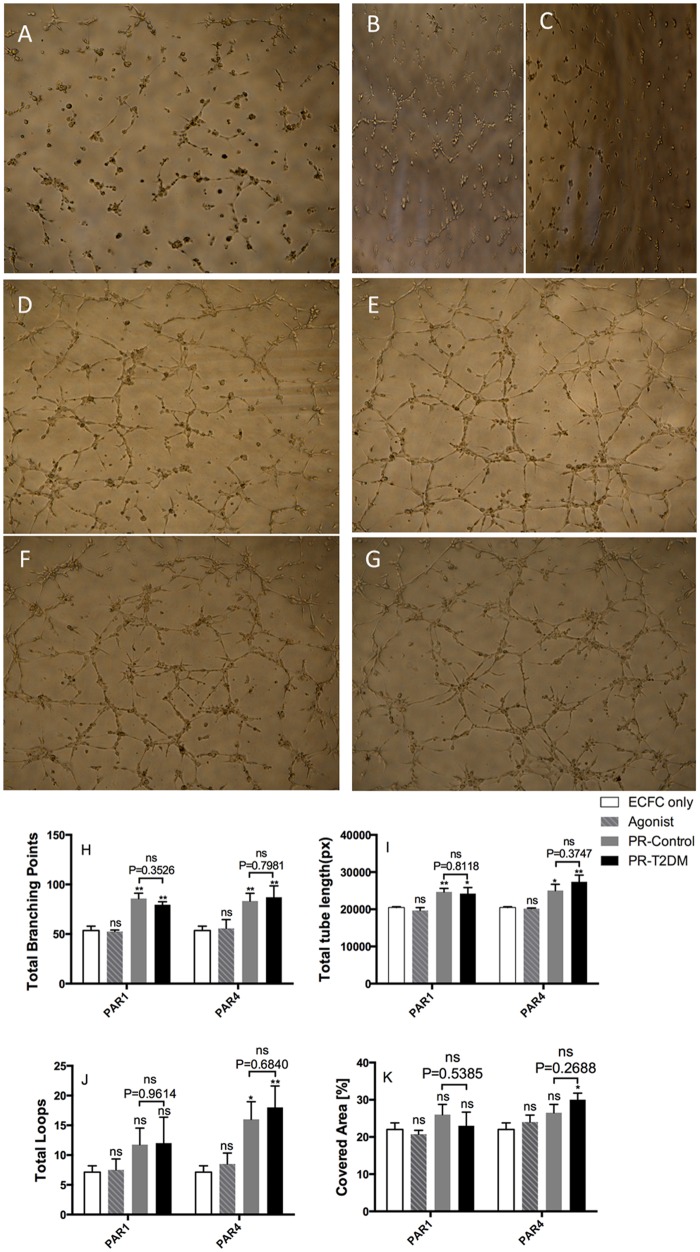
Platelet releasates enhance tube formation of ECFCs. ECFCs (1×10^4^) suspended in EBM-2 basal medium with 0.5% FBS were seeded onto Matrigel-coated wells of a 96-well plate, and incubated at 37°C for 6 h in presence of vehicle (panel A), 8 μM PAR1-AP (B), 10 μM PAR4-AP (C), 10% PAR1-PR (D and F), or10% PAR4-PR (E and G) of non-diabetic (D and E) and T2DM subjects (F and G). ECFC tube formation was observed and fotographed using an Olympus CKX41 inverted light microscope equipped with a Nikon D5100 camera. The tube formation images were subjected to WimTube tube formation image analysis. Total branch points (H) and total tube length (I) of ECFC tube formation are plotted as mean±SEM; n = 5. *P<0.05; **P<0.01; ns, not significant.

The effects of platelet releasates on ECFC tube formation was examined with an in vitro Matrigel model. Fig 3 shows that seeding of ECFCs suspended in EBM-2 basic medium with only 0.5% FBS did not show a marked formation of capillary-like tubular structures after 6 h culture (panel A), and that the same was true in the wells containing water-soluble PAR1-AP (8 μM; panel B) and PAR4-AP control (10 μM; panel C). The latter indicates that PAR activating peptides per se at those concentrations, which were present in the platelet releasates used in the experiments, had limited influences on ECFC tube formation. Supplementation of 10% PR from either control subjects (D and E) or T2DM patients (F and G) enhanced tube formation of ECFCs. Moreover, the enhancements were observed both with PAR1-PRs (D and F) and PAR4-PRs (E and G).

When capillary-like tubular structures of ECFCs were quantified using WimTube tube formation image analysis, it was found that PAR1-PRs from both non-diabetic controls and T2DM patients similarly increased the total branch points (H) and total tube length (I) of ECFC tube formation, as compared to those obtained without supplementation of platelet releasates. Similar to PAR1-PRs, PAR4-PR supplementation also markedly increased the total branch points and the total tube length of ECFC tube formation. Moreover, the enhancements of total branch points and tube length of ECFC tube formation by platelet releasates from T2DM patients were similar to those from non-diabetic controls.

### Platelets enhanced tube formation of endothelial colony forming cells

We have recently demonstrated that platelets can enhance the angiogenic activities of ECFCs through direct cell-to-cell contact, i.e., via membrane components [14]. To investigate if there is an alteration of platelet contact-dependent enhancement of ECFC angiogenic activities in T2DM patients, platelets were isolated from patients and controls, and co-incubated with ECFCs on a Matrigel-coated plate. The capillary network formation was then observed, and five 10x fields of each well were recorded further analysis.

**Fig 4:**
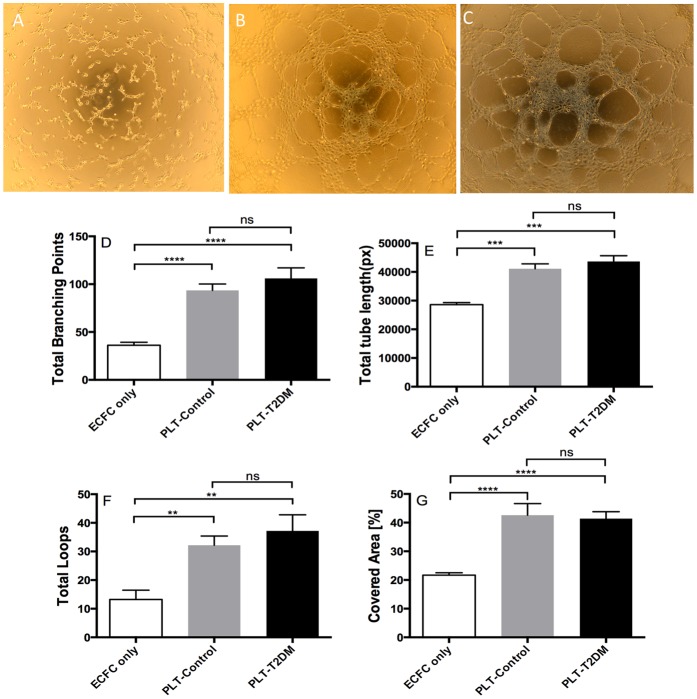
Platelets per se enhance tube formation of ECFCs. ECFCs (1×10^4^) suspended in EBM-2 basal medium with 0.5% FBS were seeded onto Matrigel-coated wells and incubated in presence of vehicle or platelets (ECFC:platelet ratio at 1:200) at 37°C for 6 h. ECFC tube formation was observed and fotographed using an Olympus CKX41 inverted light microscope equipped with a Nikon D5100 camera. The images presented are ECFC tube formation without platelets (panel A), with control platelets (B), and with T2DM platelets (C). Parameters of tube formation were analysed using WimTube tube formation image analysis. Total branch points (D), total tube length (E), total loops (F), and covered area (G) of ECFC tube formation are plotted as mean±SEM; n = 5. **P<0.01; ***P<0.001; ****P<0.0001; ns, not significant.

Fig 4A shows that ECFCs incubated alone on Matrigel-coated surface had limited capillary-like network formation. In contrast, the presence of platelets from either control (panel B) or T2DM subjects (C) markedly enhanced ECFC tube formation. The enhanced tube formation was seen as a denser capillary-like network and wider tubular structures. Hence, supplementation of platelets clearly increased the total branch points (D), total tube length (E), total loops (F), and area coverage (G) of ECFC tube formation. Moreover, the enhancements on these parameters did not differ between non-diabetic and diabetic platelets.

## Discussion

The present study investigated if diabetic platelet dysfunction would lead to altered platelet angiogenic activities in T2DM patienets. We found that platelet activation induced more marked elevation of platelet P-selectin expression (a marker of platelet secretion) and annexin V binding (an indicator of platelet phosphotidylserine exposure/procoagulant activity) in T2DM patients than in non-diabetic controls. However, we found that PAR1 or PAR4 stimulation induced similar platelet releases of angiogenic regulators in both control and T2DM subjects, and that angiogenic activities of platelets from mild T2DM patients were not changed either, as supplementation of either platelet releasates or washed platelets from non-diabetic and diabetic subjects enhanced ECFC tube formation similarly.

It is well known that platelets from diabetic patients are hyperreactive [2, 4]. Thus, the present study showed that platelet stimulation induced more marked elevation of platelet P-selectin expression, suggesting intensified platelet secretion upon activation. Importantly, our work brought new evidence that platelet activation provoked a quicker and stronger rise of platelet annexin V binding in T2DM patients. The findings indicate that diabetic platelets had a more rapid and more intense exposure of aminophospholipids (predominantly phosphotidylserine), which is a key component of platelet procoagulant activities and constitutes the prothrombinase complex together with FXa, FVa, and calcium. We have previously demonstrated that diabetic platelets are hypercoagulant, as evidenced by a more rapid burst of thrombin generation and a shortened clotting time of recalcified platelet rich plasma in T2DM patients than in non-diabetic controls [23]. Thus, the present findings reveal us a mechanism underlying hypercoagulant activities of diabetic platelets. Phosphotidylserine exposure of platelets is controlled by the balance of translocase and scramblase activities [24]. Our data imply that pathophysiological changes of T2DM have disrupted enzyme activity balance between translocase and scramblase during platelet activation, either by attenuating translocase activity, potentiating scramblase activity, or both.

Platelets have emerged as an important player in angiogenesis and tissue regeneration [25, 26]. This has prompted us to generate the hypothesis that impaired tissue regeneration, often manifested as a cumbersome wound healing, in diabetic patients may be attributed to the alterations of platelet angiogenic activities in T2DM patients. Notably, recent studies by others and us demonstrated that platelets store pro-angiogenic and anti-angiogenic regulators in different α-granules, and that PAR1 stimulation prompts pro-angiogenic, whilst PAR4 stimulation favors anti-angiogenic regulator release of platelets [8, 10, 11], albeit there are still debates about the theory [27, 28]. The present study compared PAR1-inducd and PAR4-induced angiogenic regulator release between non-diabetic and T2DM subjects, aiming to reveal if T2DM may enhance anti-angiogenic or/and reduce pro-angiogenic regulator release of platelets. We have monitored VEGF and PDGF-BB, both of which are well recognized as potent proangiogenic regulators [29, 30], as well as TSP-1 and PF4, which are well-established negative regulator of angiogenesis [31–33]. Contrasting to our assumption, we found that PAR1 and PAR4 stimulation induced similar angiogenic regulator release in T2DM and control subjects. Even more surprisingly, we found that PAR1 or PAR4 stimulation did not provoke differential platelet releases of pro- or anti-angiogenic regulators, as shown in our earlier report [10]. The likely explanation for the discrepancy is because the inhibiting cocktail, including aspirin, hirudin, and apyrase and for preventing positive feedbacks of platelet activation, was omitted during the present preparation of platelet releasates. We skipped the cocktail because the releasates would be used for ECFC culture, and we assumed that those inhibitors would complicate ECFC cultrue. However, these unexpected findings highlight the importance of positive feedbacks of platelet activation in the final readouts of platelet secretion, in which platelet released/generated ADP, thromboxane A_2_ and thrombin, secondary to the primary stimulus by PAR1-AP or PAR4-AP, may work in concert with the primary agonist to induce a general platelet secretion and subsequently mask the differential release of angiogenic regulators. Our observation may also provide a hint for the current controversy in the literature of platelet angiogenic regulator secretion that suports either differential release [8, 10, 12], activation intensity-dependent release [27], or non-differential release [28]. Nevertheless, as the result of similar platelet releases of pro- or anti-angiogenic regulators by non-diabetic and T2DM subjects and by PAR1- and PAR4-stimulation, those platelet releasates were found to enhance ECFC tube formation similarly.

We have recently reported that direct cell-cell contact via platelet tetraspannin CD151-ECFC integrin α6β1 interaction contributes importantly to platelet-enhanced ECFC angiogenic activities [14], as evidenced by that fixed platelets (i.e., no platelet secretion) partially retain angiogenesis enhancing effects, and that CD151 and α6β1 blockade attenuate the effects [14]. The present study confirmed that platelet co-culture markedly enhances ECFC tube formation, and showed that the enhancements by non-diabetic and diabetic platelets were similar. Together with our results showing that angiogenic regulator releases from diabetic and non-diabetic platelets were similar (Fig 2), and that supplementation of platelet releasates from patients and controls had identical effects on ECFC tube formation (Fig 3), these three pieces of evidence indicate that platelet α-granule contents/release of angiogenic regulators and platelet membrane components involved in platelet-enhanced angiogenesis (e.g., CD151) are unaltered in well controlled/mild T2DM patients. Hence, it may also be hypothesized that disturbed platelet angiogenic activities could only be seen in advanced/poorly controlled T2DM patients, and that the dysfunctions could happen secondary to a poor blood glucose control. As blood glucose and HbA1c of the present T2DM patients were controlled on decent levels, our data may suggest that a good glucose control is beneficial for maintaining platelet angiogenic function, despite the presence of diabetic platelet hyperreactivities.

Taken together, platelets are hyperreactive in T2DM. However, as compared to age- and gender-matched non-diabetic controls, angiogenic activities of platelets remain unaltered in mild T2DM patients.
